# The anhepatic phase extended by temporary portocaval shunt does not affect anesthetic sensitivity and postoperative cognitive function: A case–control study

**DOI:** 10.1097/MD.0000000000005654

**Published:** 2016-12-09

**Authors:** Young Gon Son, Sung Hye Byun, Jong Hae Kim

**Affiliations:** a Docheon-myeon Branch Office of Changnyeong-gun Community Health Center, Changnyeong-gun, Gyeongsangnam-do; b Department of Anesthesiology and Pain Medicine, School of Medicine, Catholic University of Daegu, Daegu, Republic of Korea.

**Keywords:** bispectral index monitor, desflurane, liver transplantation, living donors, neuropsychological test

## Abstract

Temporary portocaval shunt (TPCS) prolongs the duration of the anhepatic phase, during which anesthetic sensitivity is highest among the 3 phases of living donor liver transplantation (LDLT). Cognitive dysfunction has been associated with increased anesthetic sensitivity and poor hepatic function. Therefore, we assessed anesthetic sensitivity to desflurane and perioperative cognitive function in patients undergoing LDLT, in whom the duration of the anhepatic phase was extended by TPCS to test the hypothesis that the prolonged anhepatic phase increases anesthetic sensitivity and causes postoperative cognitive decline.

This case–control study was conducted in 67 consecutive patients undergoing LDLT from February 2014 to January 2016. Anesthesia was maintained at a 0.6 end-tidal age-adjusted minimum alveolar concentration of desflurane. The bispectral index (BIS) was maintained at less than 60 and averaged at 1-minute intervals. The mini-mental state examination (MMSE-KC) was performed 1 day before and 7 days after the LDLT. All parameters were compared between the patients undergoing TPCS (TPCS group) and the remaining patients (non-TPCS group).

TPCS was performed in 16 patients (24%). TPCS prolonged the duration of the anhepatic phase (125.9 ± 29.4 vs 54.9 ± 20.5 minutes [mean ± standard deviation], *P* < 0.0001). The averaged BIS values during the 3 phases were comparable between the 2 groups. No significant interval changes in the averaged BIS values were observed during the 3 consecutive phases. Similarly, there were no significant differences in MMSE-KC score assessed 1 day before and 7 days after LDLT between the 2 groups. The preoperative MMSE-KC scores were unchanged postoperatively in the 2 groups.

The extension of the anhepatic phase did not affect anesthetic sensitivity and postoperative cognitive function.

## 1 Introduction

Temporary portocaval shunt (TPCS) has been used in critically ill patients with toxic liver syndrome due to fulminant hepatic failure, severe hepatic trauma, primary nonfunction of a liver graft, or eclampsia, who require deceased donor liver transplantation in a short time.^[1–3]^ By maintaining portal venous flow, TPCS prevents pathophysiologic events caused by the interruption of portal venous flow for total hepatectomy, such as hemodynamic instability due to a reduction in venous return to the heart, intestinal edema resulting from congestion of the splanchnic bed, impairment of renal function, and aggravation of reperfusion syndrome.^[4–6]^ Today, TPCS is performed not only in deceased donor liver transplantation but also in living donor liver transplantation (LDLT).^[5,7]^ However, TPCS significantly prolongs the duration of the anhepatic phase.^[5]^ An anhepatic phase lasting for more than 100 minutes was found to be associated with primary nonfunction or initially poor function of a liver graft.^[8]^ Since patients with severe liver disease present with poor neuropsychological performance,^[9]^ the absence of the liver during the anhepatic phase would impair cognitive function to a greater extent. In addition, the anhepatic phase influences sensitivity to anesthetics. The dose of anesthetic required to maintain consistent anesthetic depth was lower during the anhepatic phase than during the other 2 phases.^[10–12]^ Because elderly patients with cognitive impairment are more sensitive to anesthetics than those without cognitive impairment,^[13]^ there might be a close relationship between anesthetic sensitivity and cognitive function. Therefore, we evaluated the anesthetic depth achieved by administration of a constant end-tidal minimum alveolar concentration (MAC) of volatile anesthetic during the 3 phases and the postoperative cognitive function in recipients undergoing TPCS, which prolongs the anhepatic phase, to test the hypothesis that extending the anhepatic phase by TPCS enhances sensitivity to anesthetics, leading to the impairment of postoperative cognitive function.

## 2 Methods

### 2.1 Patients

The consecutive recipients undergoing LDLT from February 2014 to January 2016 were enrolled in this prospective, observational case–control study. The Institutional Review Board of Daegu Catholic University Medical Center approved this study before it began, and all patients provided written informed consent. The inclusion criterion was liver cirrhosis regardless of the concurrent presence of hepatocellular carcinoma, whereas the exclusion criteria were alcoholic liver cirrhosis, acute liver failure, a history of central nervous system disease including hepatic encephalopathy, use of psychoactive drugs or alcohol within 6 months before and during the study period, flapping tremor affecting handwriting performance necessary for assessing constructional praxis, difficulty in communicating with medical personnel, bispectral index (BIS) values greater than 60 under the predetermined end-tidal MAC of desflurane during general anesthesia, reoperations performed within the study period, failure to wean from mechanical ventilation within 24 hours of arrival in the surgical intensive care unit (ICU) after surgery, and patient refusal.

### 2.2 Anesthesia protocol

Upon arrival in the operating room, monitoring of noninvasive arterial blood pressure, electrocardiography, and peripheral oxygen saturation was initiated. A BIS quatro sensor (Covidien Ltd., Mansfield, MA) and Adult SomaSensor (Covidien Ltd.) connected to a BIS VISTA (BISx Revision 1.13, Covidien Ltd.) and an INVOS Cerebral/Somatic Oximeter (Covidien Ltd.), respectively, were placed on the patient's forehead according to the manufacturer's instructions. After the commencement of continuous infusion of remifentanil (0.05–0.1 μg/kg/min), the right radial artery was catheterized for arterial blood sampling at 1-hour intervals and real-time monitoring of blood pressure until catheterization of the right femoral artery, which allows for monitoring of central arterial blood pressure. Then, general anesthesia was induced with 1.5 to 2.0 mg/kg of propofol, and endotracheal intubation was facilitated by 1.0 mg/kg of rocuronium, which was continuously infused at a rate of 1.0 mg/kg/h 30 minutes after the induction of anesthesia until the end of the surgery. A catheter from a Flotrac sensor (Edwards Lifescience, Irvine, CA) connected to an EV1000 monitor (Edwards Lifesciences) was inserted into the right femoral artery for continuous monitoring of central arterial blood pressure, cardiac index, stroke volume index, and stroke volume variation. The right femoral vein was cannulated to monitor the changes in inferior venal caval pressure due to surgical manipulation of the liver. Catheterization of the right internal jugular vein was performed using a PreSep Central Venous Oximetry Catheter (Edwards Lifesciences) connected to an EV1000 monitor for continuous monitoring of central venous pressure and oxygen saturation. Arterial oxygen and carbon dioxide tensions were maintained between 150 and 200 mm Hg and between 35 and 40 mm Hg, respectively, by adjusting the fraction of inspired oxygen, tidal volume, and respiratory rate. All patients were admitted to a surgical ICU after surgery and were extubated within 24 hours of arrival in the ICU.

### 2.3 Maintenance of general anesthesia using desflurane

Anesthesia was maintained by administering desflurane following endotracheal intubation. Throughout surgery, the BIS values and arterial blood pressure were maintained at less than 60 and within 20% of the preanesthetic baseline value, respectively. Until the end of the peritoneal incision, the end-tidal concentration of desflurane and dose of remifentanil were adjusted liberally to maintain the BIS values and arterial blood pressure within the predetermined ranges. Between the peritoneal incision and closure, the end-tidal concentration of desflurane was adjusted to and maintained at an age-adjusted MAC of 0.6.^[14]^ Remifentanil infusion was terminated to record the BIS values via a constant dose of an inhalation agent in the absence of the effects of opioids on anesthesia. Age-adjusted MAC was obtained using the following equation:
 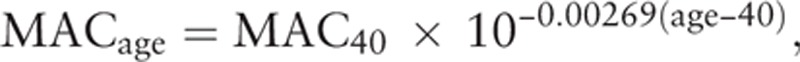


where MAC_40_ denotes the MAC value at the age of 40 years (6.6% for desflurane).^[14]^ The arterial blood pressure was maintained within 20% of the preanesthetic baseline value using inotropic or vasoactive medications without adjustment of the desflurane end-tidal concentration. However, the above anesthesia protocols were abandoned in patients excluded from the study, if an age-adjusted MAC of desflurane of 0.6 could not achieve BIS values less than 60 (leading to an increase in age-adjusted MAC), if the conventional use of antihypertensives could not control blood pressure increases due to surgical stimulation after peritoneal incision (necessitating the use of remifentanil), or if the BIS values decreased by 10 during cerebral desaturation^[15]^ (representing a decrease in regional cerebral oxygen saturation by 25% of the preanesthetic values^[16]^).

### 2.4 Collection of bispectral index data representing anesthetic sensitivity between the peritoneal incision and closure

At the end of surgery, the BIS data averaged at 1-minute intervals were retrieved from the BIS VISTA (Covidien Ltd.) using a universal serial bus memory stick. If electromyographic activity of more than 50% and/or poor signal quality represented by a signal quality index of less than 95% were noted, the BIS values were excluded from the data analysis to minimize the contamination of the BIS data by electrocautery or electromyogram artifacts. The BIS data of the preanhepatic, anhepatic, and neohepatic phases were defined as the averaged BIS values between the end of the peritoneal incision and vascular exclusion of the native liver, between the occlusion of vascular inflow of the liver and graft reperfusion, and between graft reperfusion and the beginning of peritoneal closure, respectively. Because the BIS values were collected under a constant dose of anesthetic, they could represent anesthetic sensitivity. For example, BIS values that become low at certain time points represent high-anesthetic sensitivity because deeper anesthesia is achieved at a constant dose of anesthetic.

### 2.5 Administration of mini-mental state examination in the Korean version of the Consortium to Establish a Registry for Alzheimer's Disease assessment packet

The standardized clinical and neuropsychological batteries developed for the evaluation of patients with Alzheimer's disease by the Consortium to Establish a Registry for Alzheimer's Disease were translated into the Korean language, and they had reliable performance equivalent to that of the original ones.^[17]^ Among the 8 tests from the assessment packet, the mini-mental state examination (MMSE-KC) was used to assess the cognitive function of the patients. This examination contains 19 questions with a maximum score of 30 points, and it evaluates orientation to time, orientation to place, registration, recall, attention/concentration, language, and constructional praxis, the obtainable maximum subscores of which were 5, 5, 3, 3, 5, 8, and 1, respectively (Table 1).

**Table 1:**
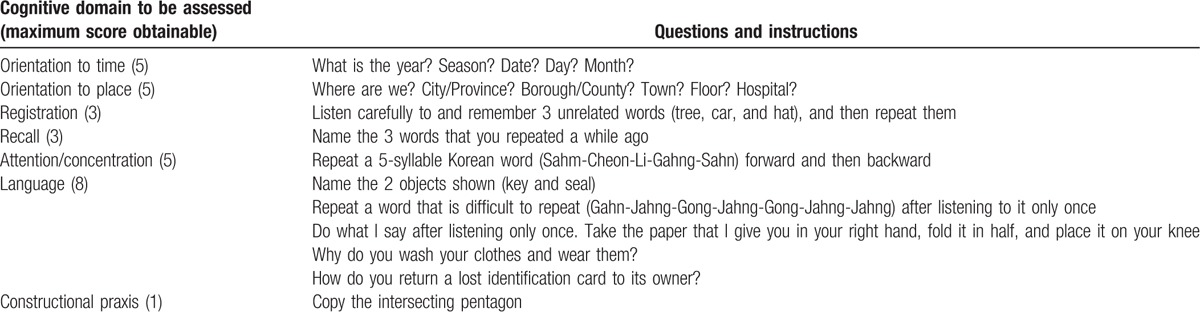
Mini-mental state examination in the Korean version of the Consortium to Establish a Registry for Alzheimer's Disease assessment packet (modified from the original version to be more applicable and comprehensible to Koreans).

The MMSE-KC was administered during the daytime in a quiet room with minimal distractions 1 day before and 7 days after surgery by a trained anesthesiologist who was blinded to the assignment of the study groups and was not involved in the anesthesia or postoperative care of the patients.

### 2.6 Surgical technique for assignment to study groups

TPCS was applied when inadvertent massive perihepatic bleeding necessitating hepatic inflow occlusion or technical difficulties resulting from perihepatic adhesions due to prior upper abdominal surgery, severe retrohepatic adhesions surrounding the inferior vena cava, or large caudate lobes that prevented retrohepatic dissection were encountered during total hepatectomy.^[5]^ Hilar dissection preceded the dissection of the retrohepatic vena cava from the native liver for TPCS, whereas full mobilization of the liver was followed by dissection of the hilar structure using the conventional extrahepatic technique. Generally, TPCS would prolong the duration of the anhepatic phase^[5]^ because the native liver is removed earlier (before the completion of the venoplasty of the liver graft) than in the conventional technique, in which total hepatectomy is performed at the end of the venoplasty.

**Figure 1:**
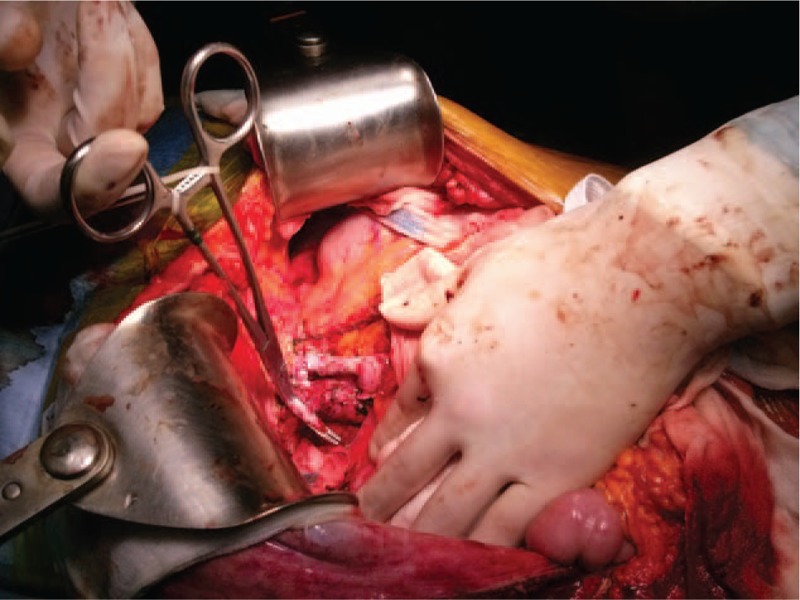
End-to-end anastomosis of the left portal vein to the middle and left hepatic vein trunk. The right portal vein was ligated, and the right hepatic vein was selectively clamped during total hepatectomy.

For TPCS, the hepatic artery, bile duct, and portal vein were dissected and clamped. The retrohepatic vena cava was preserved with selective clamping of the hepatic veins during total hepatectomy. Then, TPCS was constructed by end-to-end anastomosis of the portal vein stump to the middle and left hepatic vein trunk (Fig. 1) or by end-to-side anastomosis of the stump to the infrahepatic vena cava depending on the remaining length of the vessels. Using the piggyback technique, the donor hepatic vein was anastomosed to the orifice of the recipient hepatic vein. The portal venous flow was maintained via the TPCS until the completion of the anastomosis of the hepatic vein. For end-to-end anastomosis of the portal vein, the TPCS was closed, resulting in occlusion of the portal venous flow. Following reperfusion of the liver graft, the hepatic artery and bile duct were reconstructed. Patients undergoing TPCS were assigned to the TPCS group, and the remaining patients were assigned to the non-TPCS group.

### 2.7 Sample size estimation

The primary end point of this study was the MMSE-KC score obtained 7 days after LDLT. Using G∗power software, version 3.1.9.2 (Universität Kiel, Kiel, Germany), a sample size of 78 patients was calculated to detect a difference of 2.5 in the MMSE-KC score 7 days after LDLT between the TPCS and non-TPCS groups, achieving a statistical power of 80% at a 2-tailed alpha error of 5% under a standard deviation of 3, an unequal allocation ratio between the groups (non-TPCS:TPCS = 83:33), which is the ratio of both types of surgery performed between May 2011 and October 2013 in our institution,^[5]^ and a dropout rate of 20%, on the assumption that the Mann–Whitney *U* test would be performed due to the non-normal distribution of MMSE-KC scores in the TPCS group. A small number of patients was expected to be assigned to the TPCS group based on the number of previous cases in our institution^[5]^ and the stringent exclusion criteria.

### 2.8 Statistical analysis

The data are presented as the means ± standard deviations for normally distributed data, medians (first to third quartile) for non-normally distributed data, and numbers of patients (percentage) for qualitative data. The assumption of normality was tested using the Kolmogorov–Smirnov and Shapiro–Wilk tests. If at least 1 null hypothesis of the 2 tests was not rejected, the data were determined to be normally distributed. Univariate comparisons between the 2 groups were performed using independent samples Student *t* test for normally distributed variables, the Mann–Whitney *U* test for non-normally distributed variables, and Fisher exact test for categorical variables. If Student *t* test was used, the 95% confidence interval of the mean difference and the corresponding *P* value was presented. Repeated measures analysis of variance with post hoc paired Student *t* test using Bonferroni correction was performed for normally distributed variables to determine the significance of between- and within-groups effects and interaction between the variables (group vs phase). To determine the significant interval change in non-normally distributed variables during surgery, Friedman test with post hoc Wilcoxon signed-rank test under Bonferroni correction was used. For the comparison of within-group changes in the total sum scores and subscores of the MMSE-KC, Wilcoxon signed rank test was used. Rank analysis of covariance^[18]^ was used to compare the MMSE-KC score obtained 7 days after surgery between the groups by controlling for the covariates that were found to correlate with the score based on nonparametric Spearman correlation analysis. The statistical analysis was performed using IBM SPSS Statistics software, version 19.0.0 (IBM Corp., Armonk, NY). A 2-tailed *P* < 0.05 was considered statistically significant.

## 3 Results

**Figure 2:**
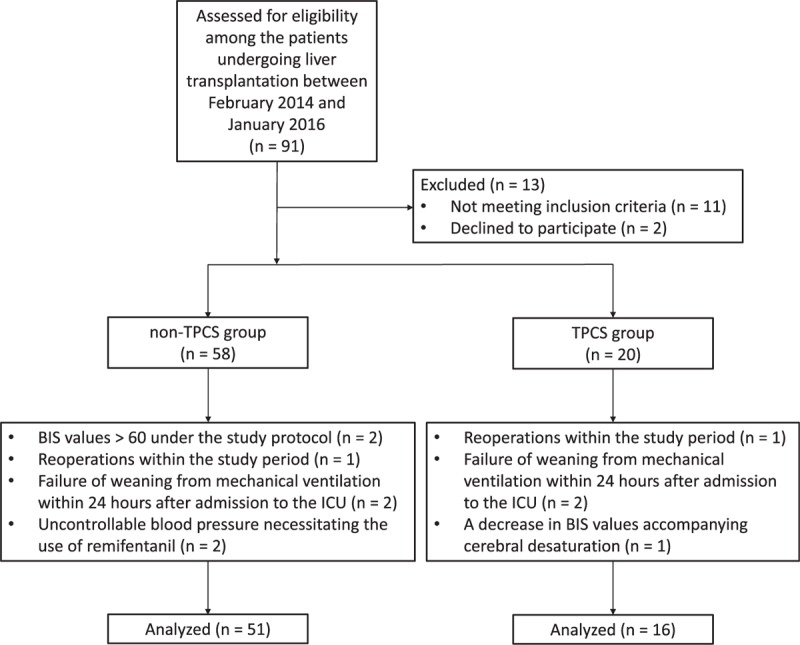
Flow diagram of patient disposition. BIS = bispectral index, ICU = intensive care unit.

Among the 91 patients undergoing liver transplantation between February 2014 and January 2016, 13 patients were excluded from the study before assignment to study groups (Fig. 2). Eleven additional patients were excluded from the analysis after assignment, resulting in 51 patients in the non-TPCS group and 16 patients in the TPCS group (Fig. 2).

There was no significant difference in the demographic data between the 2 groups (Table 2). The construction of TPCS significantly shortened the duration of the preanhepatic phase and extended the duration of the anhepatic phase, but it did not affect the duration of the neohepatic phase (Table 3). No significant changes in BIS values were observed during surgery (Table 4). The BIS values of each phase (Table 4) and the perioperative total scores of the MMSE-KC (Table 5) were comparable between the 2 groups. Even after controlling for covariates correlated with the MMSE-KC score obtained 7 days after surgery (MMSE-KC score obtained 1 day before surgery, educational period, and Model for End-stage Liver Disease Na score), a significant difference in MMSE-KC score could not be found between the 2 groups (*P* = 0.971). No significant perioperative change in the MMSE-KC total score was observed (Table 5). Although the subscore for orientation to time obtained 1 day before surgery was comparable between the 2 groups, the subscore obtained 7 days after surgery was significantly lower in the TPCS group than in the non-TPCS group (Table 5). The subscores of recall and language were significantly increased 7 days after surgery from the baseline subscores in the non-TPCS and TPCS groups, respectively (Table 5). However, no significant differences in the perioperative subscores were observed between the 2 groups (Table 5). The remainder of the subscores showed no interval changes and no differences between the 2 groups (Table 5).

**Table 2:**
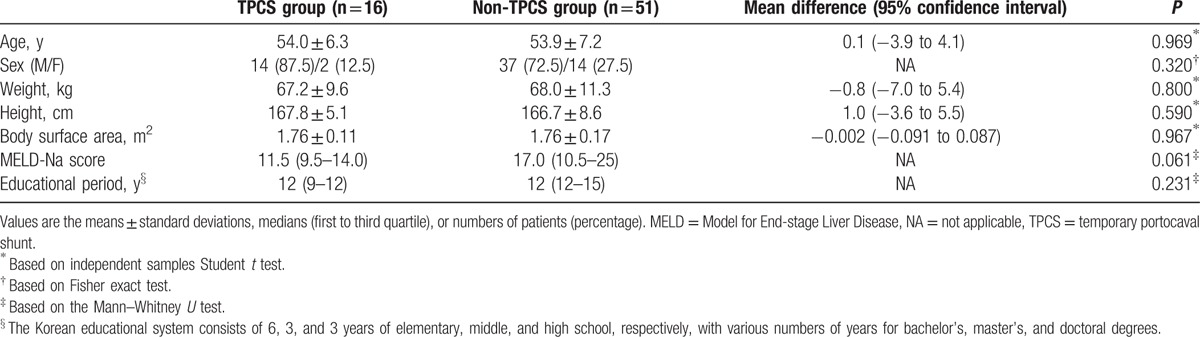
Demographic data.

**Table 3:**
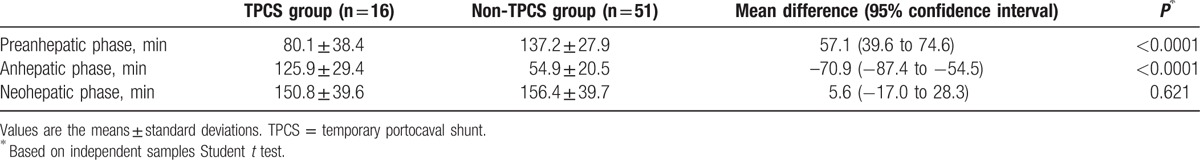
Duration of preanhepatic, anhepatic, and neohepatic phases.

**Table 4:**
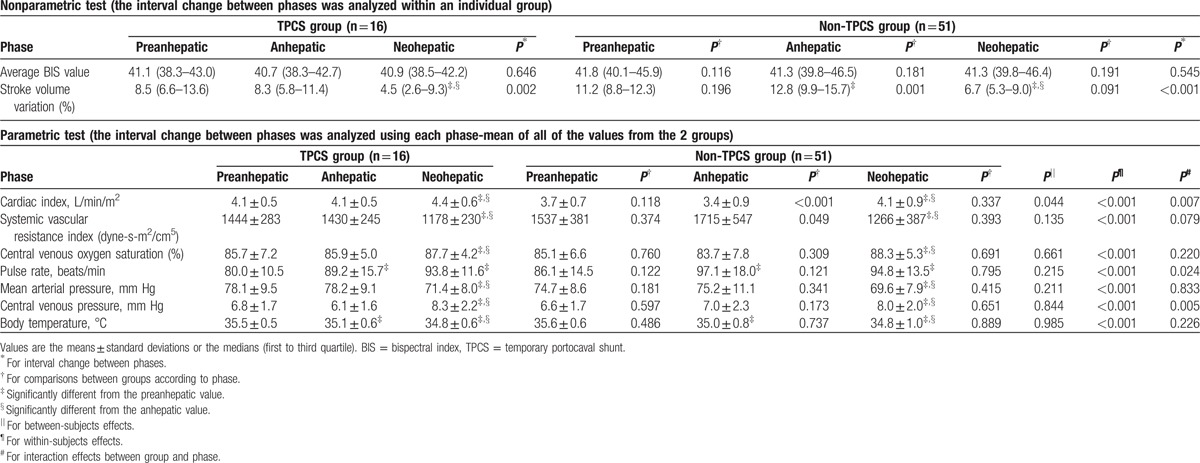
Intraoperative data.

**Table 5:**
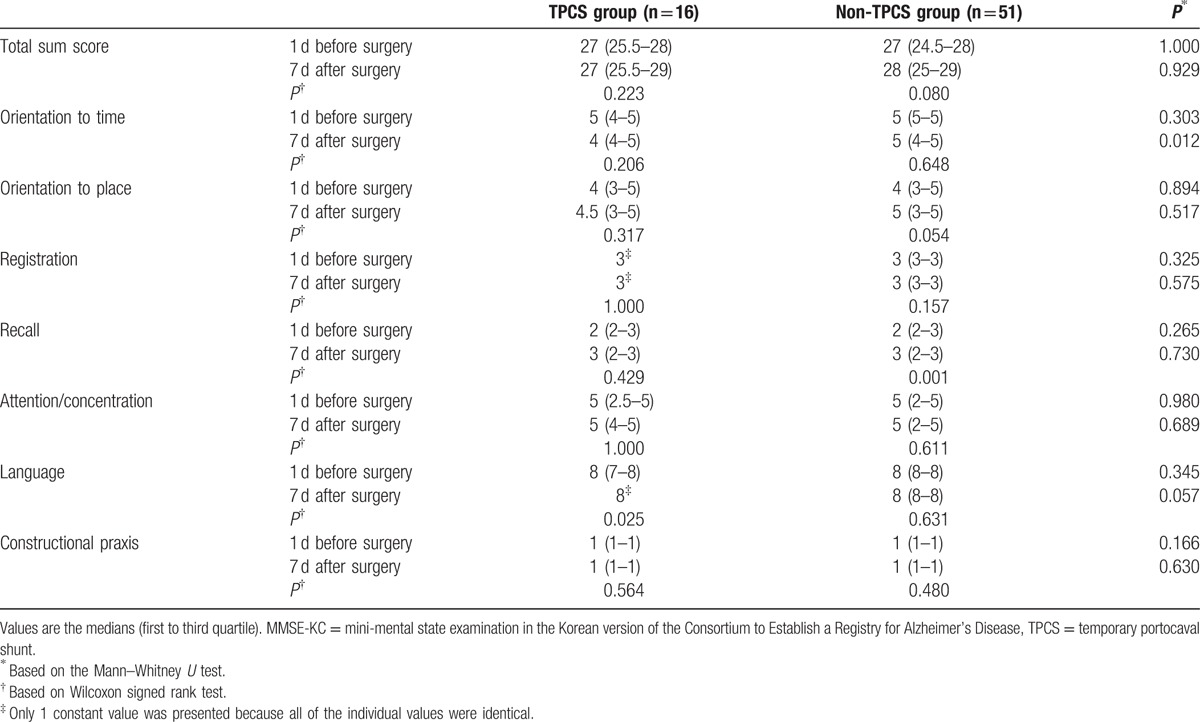
Perioperative MMSE-KC scores.

Central venous oxygen saturation, body temperature, and all systemic hemodynamic parameters, including stroke volume variation, cardiac index, systemic vascular resistance index, pulse rate, mean arterial pressure, and central venous pressure, significantly changed among the phases (Table 4). In particular, stroke volume variation was significantly lower during the anhepatic phase in the TPCS group than in the non-TPCS group (Table 4). In addition, the cardiac index during the anhepatic phase remained higher in the TPCS group than in the non-TPCS group (Table 4). These differences in stroke volume variation and cardiac index between the groups caused differences in the pattern of their interval changes among the phases (Table 4).

## 4 Discussion

The results of this study showed that prolonging the anhepatic phase by TPCS did not influence anesthetic sensitivity, as indicated by the anesthetic depth achieved using a constant dose of anesthetic and postoperative cognitive function. Furthermore, TPCS maintained intravascular volume status during the anhepatic phase better than the absence of TPCS.

The anesthetic requirements are dependent upon hepatic function. According to the results of a previous study, the end-tidal concentration of isoflurane required to maintain BIS values between 45 and 55 was lowest in end-stage liver disease patients undergoing LDLT (n = 7), intermediate in cirrhotic patients with hepatocellular carcinoma undergoing partial hepatectomy (n = 11), and highest in healthy liver donors (n = 11).^[19]^ However, the small sample size and significantly younger age in the healthy liver donor group prevented drawing a clear conclusion about the relationship between anesthetic requirements and the severity of hepatic disease. Among patients undergoing orthotopic liver transplantation, patients with more severe liver disease (Model for End-stage Liver Disease [MELD] score ≥20, n = 25) required lower end-tidal desflurane concentrations to maintain BIS values between 40 and 50 in the absence of an opioid-induced analgesic effect than patients with less severe liver disease (MELD score <20, n = 25) during the preanhepatic and anhepatic phases, but the anesthetic requirements became comparable between the 2 groups during the neohepatic phase.^[20]^ Regrettably, this study did not evaluate the interval changes in BIS values according to the 3 phases.

Compared to the preanhepatic and neohepatic phases, during which the liver remains in the recipient even though it is not functionally intact, the liver is absent during the anhepatic phase. The absence of the liver could represent the nadir of hepatic function. Because poor hepatic function is associated with lower anesthetic requirements,^[19,20]^ the anesthetic requirements are expected to be lower during the anhepatic phase than during the other 2 phases. Accordingly, the propofol target concentration required to maintain BIS values between 40 and 50,^[12]^ the end-tidal isoflurane concentration required to maintain BIS values between 40 and 55,^[11]^ and the end-tidal desflurane concentration required to maintain state and response entropy values between 40 and 60^[10]^ were lowest in the anhepatic phase than in the other phases.

However, under the above study design, which permitted a liberal change in anesthetic concentrations,^[10–12]^ expectancy bias leading to an intentional decrease in anesthetic dose during the anhepatic phase may have arisen because a broad range of anesthetic concentrations can produce a range of processed electroencephalogram values (e.g., BIS and state and response entropy) required to maintain general anesthesia in a nonlinear pattern.^[21–27]^ For this reason, the end-tidal desflurane concentration was fixed at 0.6 age-adjusted MAC, and its corresponding BIS values, which represented anesthetic sensitivity, were obtained in the present study. Unlike the results of the previous studies, which reported the highest anesthetic sensitivity in the anhepatic phase by showing the lowest anesthetic requirements to maintain the desired depth of anesthesia during this phase,^[10–12]^ our results demonstrated that there was no significant change in anesthetic sensitivity at a constant anesthetic concentration during the 3 phases in the non-TPCS group (Table 4). Despite prolongation of the anhepatic phase by TPCS, the interval change in anesthetic sensitivity remained insignificant (Table 4). Furthermore, the extended anhepatic phase did not produce a difference in anesthetic sensitivity between the 2 groups.

In addition to differences in the methods of anesthetic administration, the insignificant change in anesthetic sensitivity during the 3 phases and its insignificant difference between the 2 groups in the present study might have arisen from the difference in the duration of the anhepatic phase between ours and previous studies^[10,11]^ with data regarding the duration of the anhepatic phase. Interestingly, the average duration of the anhepatic phase extended by TPCS (125.9 minutes) was shorter than the average durations (159^[11]^ and 195^[10]^ minutes, respectively) found in previous studies, in which TPCS was not applied. The shorter duration of the anhepatic phase could not provide sufficient time to produce the effects of the anhepatic phase on anesthetic sensitivity, thereby creating the insignificant results in the present study.

The lack of significant difference in the values of processed electroencephalogram between the groups with and without postoperative cognitive dysfunction undergoing noncardiac surgery in previous studies indicated the absence of a relationship between sensitivity to anesthetics and cognitive function.^[28,29]^ Although the patients from the 2 groups received identical anesthetic administration protocols, the dose of anesthetic depended on the attending physician's discretion and the clinical situation. Were the anesthetic administration more standardized, a significant relationship between sensitivity to anesthetics and cognitive function may have been found.^[13]^ Compared to patients with normal cognitive function, patients with cognitive impairment undergoing general anesthesia using propofol had lower BIS values in the awake state, during the titration of propofol to achieve loss of reaction to verbal command, 5 minutes after the discontinuation of the anesthetic agent, and before extubation.^[13]^ The dose of propofol used to induce anesthesia was also lower in the patients with cognitive impairment than in those with normal cognitive function.^[13]^ Hence, the patients in the present study received a constant end-tidal concentration of desflurane to draw correct conclusions about the relationship between sensitivity to anesthetics and cognitive function. Nevertheless, we could not find a change in cognitive function corresponding to a change in anesthetic sensitivity in this study.

Some limitations should be considered in this study. Although the MMSE-KC is easy to administer, takes 5 to 10 minutes to complete, and has excellent test–retest and inter-rater reliability, it lacks sensitivity to minor cognitive dysfunction and presents “ceiling effects” that can result in false-negatives.^[30,31]^ In addition, repetitive assessments over short time intervals could produce learning effects in cases of mild cognitive dysfunction.^[30]^ Therefore, missing diagnoses of mild cognitive dysfunction due to ceiling and learning effects could not be avoided in our study. Aside from the duration of the anhepatic phase, which was evaluated as a factor affecting anesthetic sensitivity in this study, there might have been other factors that also affected anesthetic sensitivity, such as temperature,^[32]^ blood pressure,^[33]^ or intravascular volume status.^[34]^ Unfortunately, these factors could not be consistently maintained within normal ranges due to the unpredictable clinical situations faced during surgery (Table 4). In addition, there was a significant difference in the intravascular volume status (cardiac index and stroke volume variation) during the anhepatic phase between the 2 groups (Table 4). If the above confounding effects were significant, the results of the present study would be unreliable. However, our study design could not evaluate whether the confounding factors significantly affected the results of the present study.

Presently, further studies, which use the neuropsychological tests with a performance better than the MMSE-KC, measure clinical outcomes with longer term follow-ups (more than 1 week), and control for most confounding factors influencing anesthetic sensitivity, are warranted. In conclusion, despite the prolongation of the duration of the anhepatic phase, TPCS did not affect anesthetic sensitivity and postoperative cognitive function and even provided more favorable hemodynamic conditions during the anhepatic phase. Thus, TPCS is a safe alternative technique for LDLT.
